# Current Status of Schistosomiasis Control and Prospects for Elimination in the Dongting Lake Region of the People’s Republic of China

**DOI:** 10.3389/fimmu.2020.574136

**Published:** 2020-10-09

**Authors:** Fei-Yue Li, Xun-Ya Hou, Hong-Zhuan Tan, Gail M. Williams, Darren J. Gray, Catherine A. Gordon, Johanna Kurscheid, Archie C. A. Clements, Yue-Sheng Li, Donald P. McManus

**Affiliations:** ^1^Department of Epidemiology and Health Statistics, Xiangya School of Public Health, Central South University, Changsha, China; ^2^Department of Immunology and Diagnosis, Hunan Institute of Parasitic Diseases, Yueyang, China; ^3^Infectious Diseases Division, QIMR Berghofer Medical Research Institute, Brisbane, QLD, Australia; ^4^School of Public Health, University of Queensland, Brisbane, QLD, Australia; ^5^Department of Global Health, Research School of Population Health, Australian National University, Canberra, ACT, Australia; ^6^Faculty of Health Science, Curtin University, Bentley, WA, Australia; ^7^Telethon Kids Institute, Nedlands, WA, Australia

**Keywords:** schistosomiasis, *Schistosoma japonicum*, epidemiology, control, elimination, People’s Republic of China, Hunan Province, the Dongting Lake

## Abstract

Schistosomiasis japonica is an ancient parasitic disease that has severely impacted human health causing a substantial disease burden not only to the Chinese people but also residents of other countries such as the Philippines, Indonesia and, before the 1970s, Japan. Since the founding of the new People’s Republic of China (P. R. China), effective control strategies have been implemented with the result that the prevalence of schistosomiasis japonica has decreased markedly in the past 70 years. Historically, the Dongting Lake region in Hunan province is recognised as one of the most highly endemic for schistosomiasis in the P.R. China. The area is characterized by vast marshlands outside the lake embankments and, until recently, the presence of large numbers of domestic animals such as bovines, goats and sheep that can act as reservoir hosts for *Schistosoma japonicum*. Considerable social, economic and environmental changes have expanded the *Oncomelania hupensis hupensis* intermediate snail host areas in the Dongting lake region increasing the potential for both the emergence of new hot spots for schistosomiasis transmission, and for its re-emergence in areas where infection is currently under control. In this paper, we review the history, the current endemic status of schistosomiasis and the control strategies in operation in the Dongting Lake region. We also explore epidemiological factors contributing to *S. japonicum* transmission and highlight key research findings from studies undertaken on schistosomiasis mainly in Hunan but also other endemic Chinese provinces over the past 10 years. We also consider the implications of these research findings on current and future approaches that can lead to the sustainable integrated control and final elimination of schistosomiasis from the P. R. China and other countries in the region where this unyielding disease persists.

## Introduction

**Figure 1:**
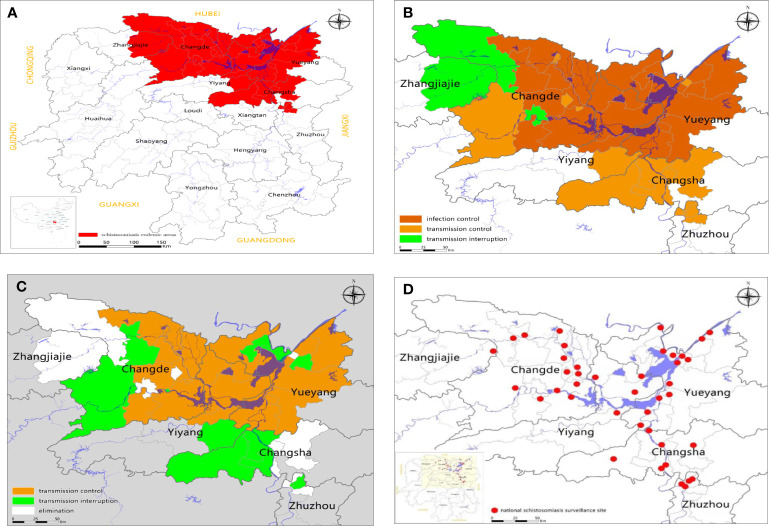
Schistosomiasis japonica endemicity over time **(A–C)** and the locations of 41 national surveillance sites (**D**: shown as red dots) in the Dongting Lake area of Hunan province, China. Brown: Infection control; Gold: Transmission control; Green: Transmission interruption; White: Elimination.

Schistosomiasis japonica is a parasitic disease that seriously endangers human health in Southeast and East Asia. In the People’s Republic of China (P.R. China) it is prevalent in the Yangtze River Basin and in 12 provinces (autonomous regions and municipalities) south of the Yangtze River area. A national census in 1959 indicated that the number of schistosomiasis patients and infected animals in the P.R. China was 11 million and 1.5 million, respectively; the number of people at risk of contracting schistosomiasis reached 100 million; and the top three provinces in terms of patient numbers were Jiangsu, Hubei, and Zhejiang (1). An area of 10, 000Km^2^ was recorded infested with *Oncomelania hupensis hupensis* in the endemic areas in the P.R. China, with Hubei, Hunan, Jiangxi, Jiangsu and Anhui provinces accounting for about 90% of these intermediate host snails (2). There has been remarkable success in control efforts over the past 70 years in endemic areas; the human infection prevalence was reduced considerably from over 60% in the 1950s to almost no human or animal infections in 2018 (3–8). Between 2008 and 2015, the schistosomiasis prevalence in humans and animals in administrative villages had decreased to below 5% and 1%, respectively (6, 7). By the end of 2017, of the 450 schistosomiasis-prevalent counties in P.R. China, 229 had reached the level of elimination, 139 were at the level of transmission interruption, with 82 having reached the level of transmission control; the number of patients with schistosomiasis had decreased to 38,000; and the number of advanced schistosomiasis cases had been reduced to 29,000 (8). Infection prevalence in most of the previously heavily infected areas dropped from 10% at the beginning of this century to less than 1%, and this low prevalence has been maintained with no active schistosomiasis transmission, and no infected snails observed by 2018 (5, 8). Nevertheless, environmental and other factors that can affect the prevalence and spread of schistosomiasis persist and the risk of infection remains in many areas (9–12). As shown by the red area in **Figure 1A**, Hunan Province is one of the regions in the P.R. China that historically has been most highly affected by schistosomiasis; endemic areas are distributed mainly in 41 counties in the Dongting Lake and surrounding areas. There were more than 600,000 patients with schistosomiasis in Hunan in the early years of Chinese liberation, including more than 10,000 patients with advanced disease (13). The marshland habitats for the oncomelanid snail hosts of *S.japonicum* in Hunan were vast, estimated at 1,766.29 km^2^ in 2004. Worryingly, these areas are increasing at a rate of 40 km^2^ annually with 80% of these being due to high silt deposition from the Yangtze River into the Dongting Lake (13). The transmission zone for *S. japonicum* along the embankment line in the Dongting Lake reaches 1,470.7 km long and has an area of 637.66 km^2^, accounting for 36.8% of the snail area outside the protective embankments in the province (13). It was projected that the giant Three Gorges Dam (TGD) across the Yangtze River would further extend the range of snail habitats, thereby increasing the potential for increased rates of transmission (14–16). **Figure 1D** shows the locations in 2017 of 41 national surveillance sites in Hunan Province. The number of individuals serologically positive by the indirect hemagglutination assay (IHA) was 3.55% (35,321/993,698) at these 41 national surveillance sites in 2017 but, paradoxically, no egg-positive faecal samples were found, no cases of acute schistosomiasis were reported, no infected animals were identified, and no schistosome-infected snails were recorded (5). In contrast, 14 subjects were identified with egg-positive fecal samples in Zejiang, Anhui and Jiangxi provinces (6). There are many floating fisher communities in Hunan Province, and they move frequently from other schistosome-endemic areas to the Dongting Lake region. Therefore, the potential for recurrent transmission of schistosomiasis in this region cannot be underestimated. The success or failure of schistosomiasis control in Hunan will impact directly on the P.R. China’s national control programme aimed at blocking transmission and eliminating schistosomiasis. In this paper, a thorough search of literature on schistosomiasis and *Schistosoma* was conducted using the Web of Science database, the Chinese online libraries of CNKI, WANFANG DATA, and VIP INFORMATION, the National Science and Technology Library, and personal archives on schistosomiasis and schistosomiasis control in the Dongting Lake region. Particular emphasis was placed on research undertaken over the past 10 years. As a result, the history and the current status of schistosomiasis control in the Dongting Lake region were systematically reviewed. We also explored the epidemiological factors contributing to *S. japonicum* transmission in order to provide insight into future approaches for control that might finally lead to the elimination of schistosomiasis from this region, other endemic areas in the P.R. China and beyond.

## Schistosomiasis in the Dongting Lake Region and the History of Its Control

*S. japonicum* eggs were found in the remains of a female corpse from the Western Han Dynasty unearthed from the Han Tomb No. 1 of Mawangdui in Changsha in 1972, indicating that schistosomiasis has been prevalent in the Dongting Lake area for at least 2,100 years (17, 18). In 1905, an American doctor (OT Logan) discovered *S. japonicum* eggs in the faeces of a young fisherman in Zhoujiadian, Changde, Hunan Province; this was the first report of a schistosomiasis case in the P.R. China (19). In 1950, the Hunan government started to investigate the distribution of schistosomiasis in the province and, as a result, adopted a comprehensive prevention strategy based on targeted snail control in transmission zones and the expansion of drug treatment to humans and animals. These measures reduced snail densities and the number of infected snails and, as a result, the schistosomiasis prevalence fell markedly (13, 20). However, due to a substantial reduction in financial support, particularly the termination of the China World Bank Loan Project (WBLP) for schistosomiasis control, control efforts declined (21). Heavy flooding in the Yangtze River Basin, the impact of global warming, and the increased mobility of villagers and the floating fisher population, also contributed to a rebound of schistosomiasis in Hunan and other endemic Chinese provinces in the early 21^st^ century. During this time, the total number of human cases in P.R. China increased from 756,762 in 1999 to 843,007 in 2003, including 1114 acute human infections (234 cases from Hunan province), compared with 513 acute cases (198 cases from Hunan province) in China in 1999. In P.R. China, 24,461 bovines were found to be egg stool-positive compared with 21,457 in 1999. An area of 2.77 Km^2^ in Hunan province was shown, for the first time, to be infested with oncomelanid snails (22, 23).

**Figure 2:**
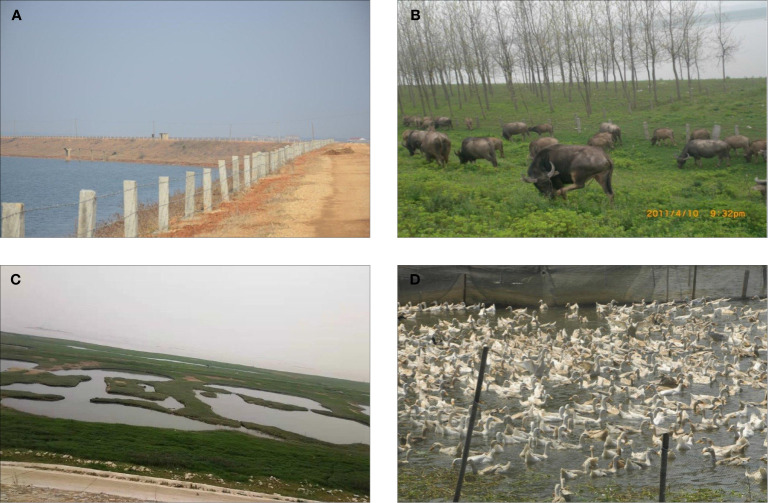
Management of snail-infested areas in the Dongting Lake area, Hunan province, P.R. China. **(A)** Fences by a lake side area. **(B)** Buffalo feeding on pasture fenced off from a lake area. **(C)** Dongting Lake area after removal of domestic animals. **(D)** Raising poultry instead of domestic livestock animals.

Since 2004, Hunan Province has implemented the Chinese national schistosomiasis control strategy for controlling the transmission of *S. japonicum* mainly through targeting domestic animals and fishermen as sources of infection. As a result, both human and animal *S. japonicum* infection prevalence was reduced to 1.46% and 2.34% in 2008 from 3.35% and 4.923% in 2003, respectively (5, 24). In 2008, as **Figure 1B** indicates, the entire province reached the level of schistosomiasis infection control and the control policy shifted from reducing the prevalence of infection to transmission control (25, 26). Since 2010, under the support of the joint action plan of Hunan Province and the Chinese Ministry of Health, an integrated schistosomiasis control strategy has been promoted and implemented. The measures applied by the health and other relevant sectors include fence closure, shoal closure, animal grazing prohibition in snail zones (**Figures 2A, B**), management of schistosomiasis reservoirs such as buffalo, cattle and sheep, education of the floating fisher population, and rigorous monitoring of transmission areas. In 2015, Hunan Province reached the level of schistosomiasis transmission control with human and animal prevalence levels decreased to 0.23% and 0.28%, respectively at the administrative village level (27–30). By the end of 2018, the number of patients with advanced schistosomiasis in Hunan Province had been reduced to 5,034 and 13 of its 41 counties had reached the level of transmission interruption with 10 reaching the level of elimination (8). **Figures 1B, C** show a comparison of the extent of schistosomiasis control around the Dongting Lake area in 2008 and 2018, respectively. Based on schistosomiasis status and the technologies available at different periods of time, three distinct control strategies were in operation in Hunan province, summarized in **Table 1**.

**Table 1:** Prevalence of schistosomiasis and the control strategies operating over different time periods in Hunan province [Refs (1, 5, 6, 8, 24)].

Period	Prevalence of schistosomiasis	Main Control Strategy	Other Strategies
**1950s**	Human infection prevalence: 20.00%–68.70%. Estimated human cases: over 600,000. Snail infested areas: >2,000km^2^.	Snail control by environmental modification.	Surveillance. Limited human drug treatment and mollusciciding.
**1980s**	Human and animal infection prevalence: 5.0%–6.0%. Estimated human cases: >270,000. Snail infested areas: >1,500km^2^.	Morbidity control by mass praziquantel chemotherapy of humans and animals.	Surveillance. Health education. Snail control with environmental modification and mollusciciding. Safe water provision and improved sanitation.
**2003**	Human and animal infection prevalence: 3.35% and 4.92%, respectively. Estimated human cases: 205,932. Acute infection cases: 198. Snail infested areas: 1,752.52km^2^.	Transmission control with integrated approaches mainly targeting human and animal sources of infection.	Surveillance. Praziquantel treatment. Health education. Snail control with environmental modification and mollusciciding. Safe water provision and improved sanitation. Control regulation law enforcement (commenced in 2006).
**2018**	No active human or animal infections. Estimated human cases: 24,986. Snail infested areas: 1,730.85km^2^.	Transmission control with integrated approaches mainly targeting human and animal sources of infection.	Surveillance. Praziquantel treatment. Health education. Snail control with environmental modification and mollusciciding. Safe water provision and improved sanitation. Control regulation law enforcement. Fishing banned in Dongting Lake for 10 years commencing in 2019.

Located in the northern part of Hunan province and on the south bank of the middle reaches of the Yangtze River, Dongting Lake is a flood basin receiving waters from the Xiang, Zi, Yuan, and Li rivers. The water system around Dongting Lake comprises a reticulated distribution network. There are a large number of shoals present harbouring considerable numbers of oncomelanid snails. The schistosomiasis-endemic area in Hunan Province is centred on the Dongting Lake Plain and is distributed radially to the west, the south, and in hilly areas in the east; it is divided into two types: marshland-type and hill-type, with the marshland-type dominating. Ninety-seven percent of the total recorded numbers of patients in Hunan province were residents of the marshland areas of Dongting Lake. Natural, ecological, social, and economic factors influence the prevalence and spread of *S. japonicum* (31–35). Changes in water level, temperature, and sand sedimentation, in combination with floods and other environmental disasters in the Yangtze River Basin present unique epidemiological characteristics that accelerate the spread of snails and intensify schistosomiasis transmission in the Dongting Lake area (36). The association between snails and several environmental factors has been reported as an inverted U-shape in the marshlands of the Dongting Lake Region (37); optimal soil moisture content, pH, temperature and land elevation in this Lake area, for snail to survival and reproduction are 58.70% to 68.93%, 6.6 to 7.0, 22.73°C to 24.23°C, and 23.5 m to 26.0 m, respectively. Snails survived less well when the levels of any of these environmental factors were either lower or higher (37). The Dongting Lake area has complex terrain with frequent human and animal interactions, high densities of infected snails and resultantly high water-infectivity to humans and animals, increasing continuing *S. japonicum* transmission. The most highly exposed group of people is fishermen (38), who live on boats and whose excrement is discharged directly into lake water resulting in long-term and extensive contamination (39). There are more than 60,000 active fishermen in the Dongting Lake area; they come from more than 10 provinces across the country and undertake numerous water-based activities in the lake throughout the year. The flooding of shoals with snails from May to September is the most frequent season for exposure of fishermen to schistosome-contaminated water (13). The annual water level in the Dongting Lake changes by about 15 meters, rising in summer and falling in winter, and the pattern of transmission is similar to that in the Poyang Lake in Jiangxi Province; unlike other provincial areas, there is continual transmission in the Dongting Lake from spring to autumn but with two distinct peaks. Coinciding with the commencement of the annual rainy season, transmission commences in April when conditions become favourable for snails as the water levels rise and the temperature increases due to the onset of spring; the first peak for *S. japonicum* transmission is from May to early July. When the water level rises to its maximum, remaining high from mid-July to September, and the risk of transmission becomes lower. The second peak commences in October, when the water level drops, and it normally ends in November (17, 40).

## Impact of the Three Gorges Dam (TGD) on *S. japonicum* Transmission in the Dongting Lake

The Three Gorges hydroelectric dam that became operational in 2003 is a considerable feat of modern engineering, providing power to a vast area, lowering the flood risk along the Yangtze, and increasing the river’s shipping capacity. Now there is growing evidence to show that the dam may have contributed to increased schistosomiasis transmission in some of the surrounding areas, but a reduction in others. With the completion of the dam, changes in the water level in the main stream of the Yangtze River and in the lower reaches of the TGD reservoir in Tongjiang Lake altered the ecological environment in the middle and lower reaches of the River. These factors may have led to changes in the distribution of snails in the Dongting Lake area (41). The potential impact of the TGD on the transmission of *S. japonicum* in the Yangtze River basin has raised concerns from researchers worldwide (42–44). Numerous studies have been carried out to forecast or assess the impact of the dam on the distribution of *O.h. hupensis* snails and the transmission of *S. japonicum* (15, 16, 45–48). A study by Han et al. (47) showed the ecological restoration process in the Dongting Lake region was fairly slow and inefficient by surveying the community structure and diversity of soil animals, including gastropod snails, obtained from three typical habitat types, i.e., wetland restored from farmland, original farmland, and original wetland; 26 species were obtained from wetland restored from farmland. In another study, a team of Chinese scientists set out to determine whether the TGD would impact negatively on the progress of elimination of schistosomiasis (15). Several natural lakes down river from the dam are endemic for *S. japonicum* and its *O. h. hupensis* intermediate host, and the Chinese team analysed 55 published research articles relating to the dam’s impact on schistosomiasis; 2/3 articles predicted an impact while 1/3 publications reported an existing impact and found, overall, that the dam has impacted (or will impact) positively towards schistosomiasis elimination (15, 49). This is due to the fact the dam has been changing the hydrology of the area by controlling natural flooding and has led to the reduced dispersal of snails. Another study on hydrological and schistosomiasis-endemic status changes, before and after the TGD was established, was conducted in 12 surveillance sites of the Hunan section of the Yangtze River (16). The results showed a number of ecological environmental factors had changed in the Dongting Lake area after the completion and operation of the TGD; the volume of annual runoff discharged into the Dongting Lake declined by 20.8%, and the annual sediment volume discharged into the lake and the mean lake sedimentation rate decreased by 73.9% and 32.2%, respectively. From 2003 to 2015, the areas with living snails and the mean density of living snails (using 0.11m^2^ frames as the units for snail sampling surveys in the field) decreased overall by 82.43% and 94.35%, respectively, with annual decreases being 13.49% and 21.29%. Moreover, the human schistosome infection prevalence had decreased from 3.35% in 2003 to 0.44% in 2015, with the overall reduction being 86.98%. Correlation analyses showed that the mean density of living snails was significantly associated with water level, as well as the mean elevation range of the marshland and the *S. japonicum* infection rate (16). Ecological environmental changes caused by the TGD were shown to correlate with the distribution of oncomelanid snails, and potentially further impacting the transmission and prevalence of schistosomiasis (48, 49). The risk of schistosomiasis transmission still exists in the Dongting Lake area and long-term monitoring is, therefore, essential. The number of days an area is inundated with water may be a key factor determining the geographical distribution of *Oncomelania* snails in Hunan Province; the favourable period of inundation for the survival of snails ranges from about 2 to 7 months with the snail density shown to be highest when flooding lasts for 4 to 5 months (48, 49).

## Transmission of *S. japonicum* to Humans From Animals

Unlike the other main human forms of schistosomiasis caused by *S. mansoni*, *S. haematobium*, and *S. intercalatum*, schistosomiasis japonica is a zoonosis with 46 species of wild and domestic animals able to harbour the infection (50, 51). This wide host range complicates control efforts as these animal reservoirs contribute considerable environmental contamination with *S. japonicum* eggs. Until recently, water buffaloes and cattle openly grazed in the snail zones in the lake-type areas in China; bovines harbour long-term infection and high infection rates, resulting in these animals being the main transmission source, contributing up to 90% of the transmission of *S. japonicum* (52–54). The daily fecal output from a water buffalo is estimated to be at least 100 times greater than that produced by a human individual (44). As result, bovines have been targeted specifically in the China national schistosomiasis control programme, recognized as one of the most successful worldwide (55). By 2018, most bovines had been removed from the majority of the schistosome-endemic areas around the Dongting Lake (**Figure 2C**) and replaced by motorised tractors for farming, although small numbers of animals remained on the marshland. A recent study determined the infection prevalence of *S. japonicum* in rodents (0–9·21%), dogs (0–18·37%) and goats (6·9–46·4%) from the area, using a combination of traditional coproparasitological techniques (miracidial hatching technique and Kato-Katz thick smear technique) and molecular methods [quantitative real-time PCR (qPCR) and droplet digital PCR (ddPCR)] (56). The study indicated a much higher prevalence in goats than previously recorded in this setting. Cattle and water buffalo, examined using the same procedures, were all found infected, demonstrating that active transmission was still occurring. Goats graze on the marshland areas of the Dongting Lake and, like bovines, they also frequently contact with water. The high level of *S. japonicum* prevalence in goats indicates that they are likely important reservoirs in schistosomiasis transmission, necessitating their inclusion as targets of control, if the goal of elimination is to be achieved in the P.R. China (56).

## Current Strategies and Progress on Schistosomiasis Control

With the decline in the number of schistosomiasis patients, the Chinese government has modified its approach for schistosomiasis control. Based on the differences in transmission modes and the endemic conditions prevailing, a joint effort with other relevant sectors has been proposed for affected areas with the scope, the focus and what to survey determined accordingly. Additionally, establishment of a sensitive and effective monitoring and early warning system is necessary to provide the solid foundation required for the elimination of schistosomiasis (57–61). In 2017, the National Health and Family Planning Commission and six other departments issued the *13^th^Five-Year Plan for the Prevention and Control of Schistosomiasis in China*, which opened up a new pathway for halting the spread of schistosomiasis in P.R. China and targeting its elimination (62). According to the requirements of this plan, and the *Plan for the Elimination of Schistosomiasis in Hunan Province (2016*–*2025)*, the local authorities are, in addition to undertaking active promotion of the procedures necessary to eliminate schistosomiasis from the province, implementing four major aspects of control comprising: population infection source control, livestock infection source control, snail control, and schistosomiasis control institutional capacity building; as well as undertaking active promotion of the procedures necessary to eliminate schistosomiasis from the province. Major successful outcomes of these control efforts have been a well organised national operating system, community participation, effective administrative involvement, and relevant technical support. The current national control strategy for the Dongting Lake area and the P.R. China as a whole employs a multi-component integrated approach with mass administration of praziquantel to humans and bovines as its cornerstone - combined with snail control through chemical mollusciciding and environmental modification; improved sanitation through the supply of safe drinking water and latrines; health education aimed at effectively increasing people’s knowledge of schistosomiasis; and removal of cattle, water buffaloes and sheep from all endemic areas (4, 7, 25, 63, 64). One area that highlights the effectiveness of this approach is Junshan District, in the Dongting Lake region; Li et al. (63) showed that the multi-component control tactic resulted in a decrease in the prevalence of schistosome infection in residents from 3.44% in 2006 to zero in 2016 after removal of all animals in 2013. As a result of the exclusion of bovines and sheep from the beaches in the endemic areas, the income of the local farmers declined; consequently, the government provided support to these farmers to raise poultry to replace the livestock animals (**Figure 2D**).

In the P.R. China, a dramatic demographic transition has coincided over the last 40 years with equally intense economic reforms and rapid economic growth. The Chinese rural economy has undergone considerable structural changes with young people from rural areas migrating to cities for employment, to be closer to friends or to reside with other family members. Consequently, the population in the schistosomiasis-endemic areas has decreased considerably and the age-structure of the general population has changed. In many rural villages, schoolchildren and older aged villagers predominate. As a result, the decreased schistosomiasis prevalence can be partly attributable to fewer at risk individuals, such as farmers contacting with infested water, and improvements in the prevailing economic and sanitation conditions (6).

## Future Perspectives and Challenges

Although the path towards schistosomiasis control and prevention in Hunan Province has resulted in considerable achievements over the past several decades, a number of problems and challenges remain. Critical factors that maintain the transmission of schistosomiasis are still current, and these will likely impact upon the eventual goal of elimination. Firstly, the schistosomiasis-endemic area around the Dongting Lake is vast, it has a wide snail distribution range, it is a complex environment, and the spread of snails into both existing and new locations remains a constant concern. In 2017, 646 administrative villages in Hunan Province harboured oncomelanid snails in an area covering 1,731.29 km^2^, accounting for nearly half of the total snail area for the whole of the P.R. China (8). Secondly, the source of schistosome infection still exists (56), although infection prevalence has been reduced. Currently, there is some disagreement over the use of shoal farmland in the endemic areas to develop aquaculture, and the schistosomiasis control measures involving the removal of domestic livestock and the banning of grazing on the shoals. Furthermore, the livelihood of the itinerant fishermen is highly dependent on the Dongting Lake. The introduced management measures prohibiting the grazing of animals and the prevention and control measures involving fishermen remain difficult to implement long-term. Currently, some livestock and fishermen still frequent the snail zones, and the risk of schistosomiasis infection remains a constant threat (65, 66). Thirdly, increased migration of people to and from cities has led to an increase in the mobility of potential schistosomiasis infection sources and in the number of individuals entering some of the endemic areas (67). As the schistosomiasis control strategy in the P.R. China moves from disease control to elimination, improved surveillance and monitoring assessment activities will play a key role in detecting new infections and in identifying new areas of transmission (68–70). Additional prevention and control measures and increased government support will be required to maintain the decline in schistosomiasis cases and to reduce any potential factors that could lead to the re-emergence of the infection and to ensure its elimination (62, 70–72). In accordance with the overall deployment of the Special Project of Three-year Tactical Action Plan for Local Endemic Disease Prevention and Control (2018–2020) (73), Hunan Province will further integrate departmental resources, strengthen monitoring systems and continue to implement integrated prevention strategies based on local conditions and published control classification guidelines in order to achieve schistosomiasis elimination by 2030. If the transmission blocking and elimination targets are not achieved, the control of infectious sources including the comprehensive management of existing snail environments will need to be strengthened, *S. japonicum* transmission risk assessment should be strictly adhered to, and monitoring and early warning systems should be improved. In addition, livestock containment, rigorous surveillance methods and the availability of more sensitive diagnostics will be paramount to achieve success (69, 74). Vaccination of bovines and other reservoirs represents an important intervention to the schistosomiasis armory and may represent the best hope for achieving eventual elimination but, despite some encouraging recent progress (75, 76), no such vaccine has been developed to an acceptable level for wide-scale use.

## Author Contributions

F-YL and X-YH participated in literature searches, and data extraction; F-YL drafted the manuscript; X-YH, H-ZT, Y-SL, GMW, DJG, CAG, JK, ACAC, and DPM critically reviewed the manuscript for its intellectual content. All authors contributed to the article and approved the submitted version.

## Funding

China Hunan Provincial Science and Technology Department (project No.2017WK2073) and the National Health and Medical Research Council of Australia (ID: APP1160046, APP1037304 and APP1098244). DPM is a NHMRC Senior Principal Research Fellow and Senior Scientist at QIMR Berghofer Medical Research Institute.

## Conflict of Interest

The authors declare that the research was conducted in the absence of any commercial or financial relationships that could be construed as a potential conflict of interest.
